# Cysts in Periradicular Region of Deciduous Molars in Mixed Dentition: Retrospective Study of Five Cases

**DOI:** 10.5005/jp-journals-10005-1273

**Published:** 2015-02-09

**Authors:** Varsha Sunil Manekar, Ankush Chavan, Kavita Wadde, Vishal Dewalwar

**Affiliations:** Associate Professor and Head, Department of Oral and Maxillofacial Surgery, Government Dental College and Hospital, Mumbai, Maharashtra, India; Assistant Professor, Department of Oral and Maxillofacial Surgery, Government Dental College and Hospital, Mumbai, Maharashtra, India; Assistant Professor, Department of Oral and Maxillofacial Surgery, Government Dental College and Hospital, Mumbai, Maharashtra, India; Assistant Professor, Department of Oral and Maxillofacial Surgery, Government Dental College and Hospital, Mumbai, Maharashtra, India

**Keywords:** Cyst, Radicular cyst, Dentigerous cyst, Deciduous molar, Developing premolar.

## Abstract

The cyst in mixed dentition stage cause expansion of buccal cortex, displacement of teeth and may present as case of infection. The cyst in periradicular region of deciduous molar are of frequent occurrence. The differential diagnosis of this lesion is radicular cysts of deciduous molar: developmental or infammatory dentigerous cyst of corresponding unerupted premolar. After going through the available literature of radicular cyst of deciduous molars and dentigerous cysts of developing premolars in mixed dentition we studied the five cases of cyst in periradicular region of deciduous molars in mixed dentition retrospectively for the diagnostic dilemma of radicular cyst verses dentigerous cyst. In conclusion, we can set some criteria for the diagnosis which is particularly important for treatment and for conservation of developing premolar.

**How to cite this article:** Manekar VS, Chavan A, Wadde K, Dewalwar V. Cysts in Periradicular Region of Deciduous Molars in Mixed Dentition: Retrospective Study of Five Cases. Int J Clin Pediatr Dent 2014;7(3):229-235.

## INTRODUCTION

The cyst in periradicular region of deciduous molars in mixed dentition present with the symptoms of swelling, pus discharge, pain and over retained teeth. The expansion of buccal cortex and displacement of corresponding developing premolar is a common presentation. These cysts can be radicular cysts of deciduous molars or; developmental or infammatory dentigerous cysts of corresponding premolar or supernumerary tooth bud. In case of carious or endodontically treated deciduous molar, there is possibility of radicular cyst of deciduous molars or inflammatory dentigerous cyst of corresponding premolars. After going through the available literature of radicular cyst of deciduous molars and dentigerous cysts of premolars we studied the five cases of cyst in periradicular region of deciduous molars in mixed dentition treated in our department for the diagnostic dilemma of radicular cyst *vs* dentigerous cyst. This is particularly important for treatment, prevention of localized malocclusion and conservation of premolar toothbud. Aim – Retrospective study of cases of cyst in periradicular region of deciduous molars in mixed dentition. Objective – To set criteria for diagnosis of radicular cyst *vs* dentigerous cyst. Inclusive criteria-the cases diagnosed as cyst in posterior jaw of mixed dentition with radiolucency in periradicular region of deciduous molars with corresponding premolar bud in lesion. Cases reported to Department of Oral and Maxillofacial Surgery, at Government Dental College and Hospital, Mumbai were studied. There were five patients with diagnosis of infected cyst with deciduous molar region in mixed dentition; they were comparatively studied for their clinical presentation, radio-graphic presentation, and differential diagnosis, outcome of treatment and for criteria of definitive diagnosis of radicular cyst of deciduous molar or dentigerous cyst of developing premolar.

## OBSERVATIONS

Table 1 shows the case details of clinical and radiographic presentation. Age range of patients is 6 to 13 years, with mean age of 8.8 years with three female and two male patients. The lesion involves maxilla in one case and mandible in four cases. In one case lesion is with first deciduous molar and in four cases with second deciduous molar. All cases are of similar size. Four cases show moderate expansion and one case had severe expansion.

**Fig. 1:**
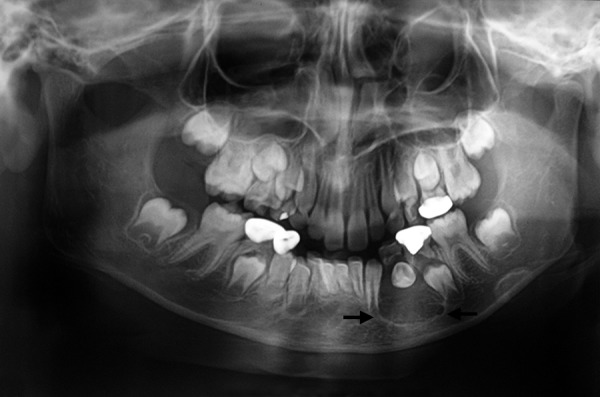
Preoperative OPG (Case 1)

**Fig. 2:**
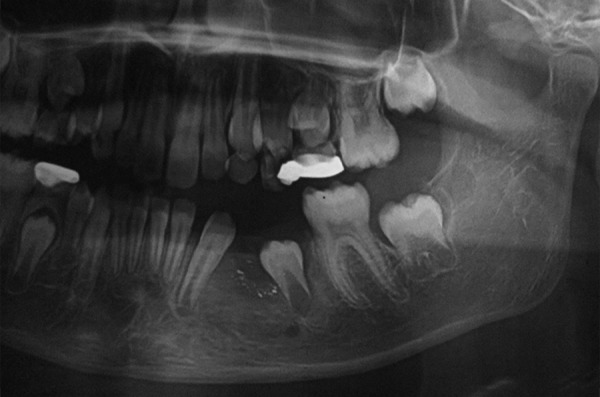
Postoperative OPG (Case 1)

**Fig. 3:**
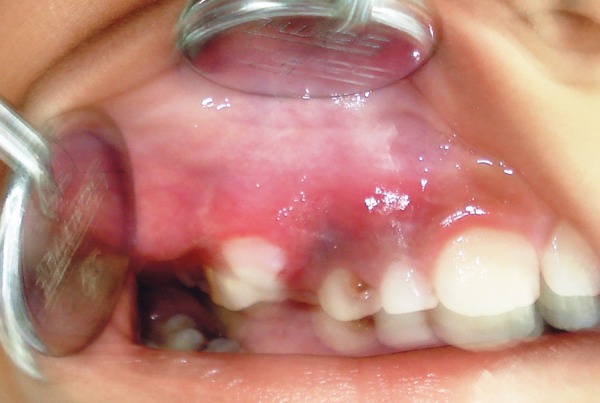
Clinical preoperative image (Case 2)

**Fig. 4:**
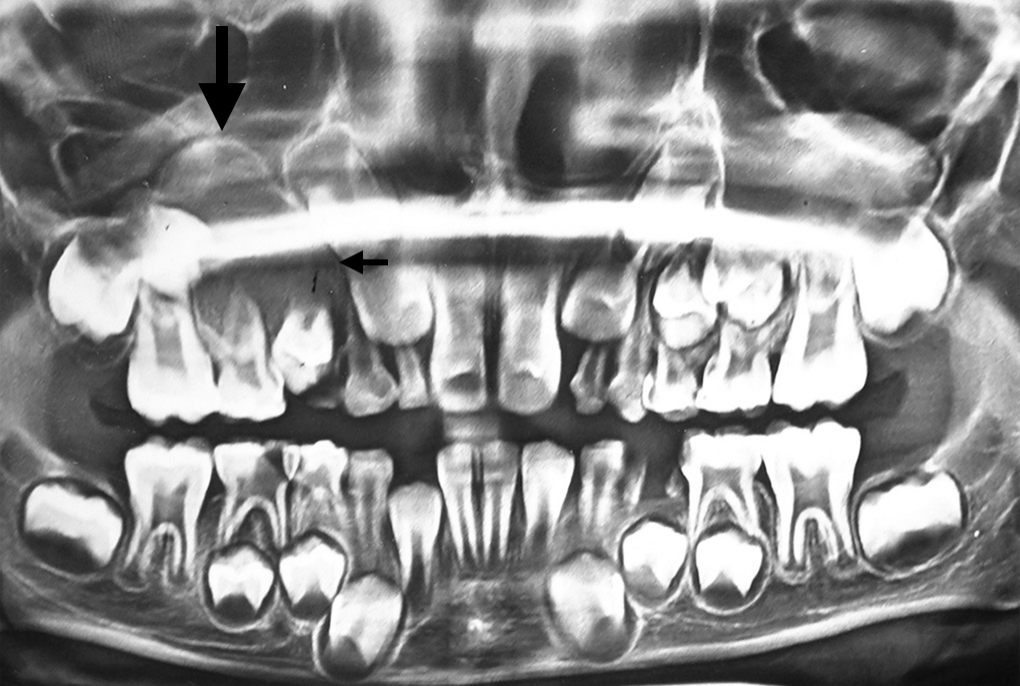
Preoperative OPG (Case 2)

**Fig. 5:**
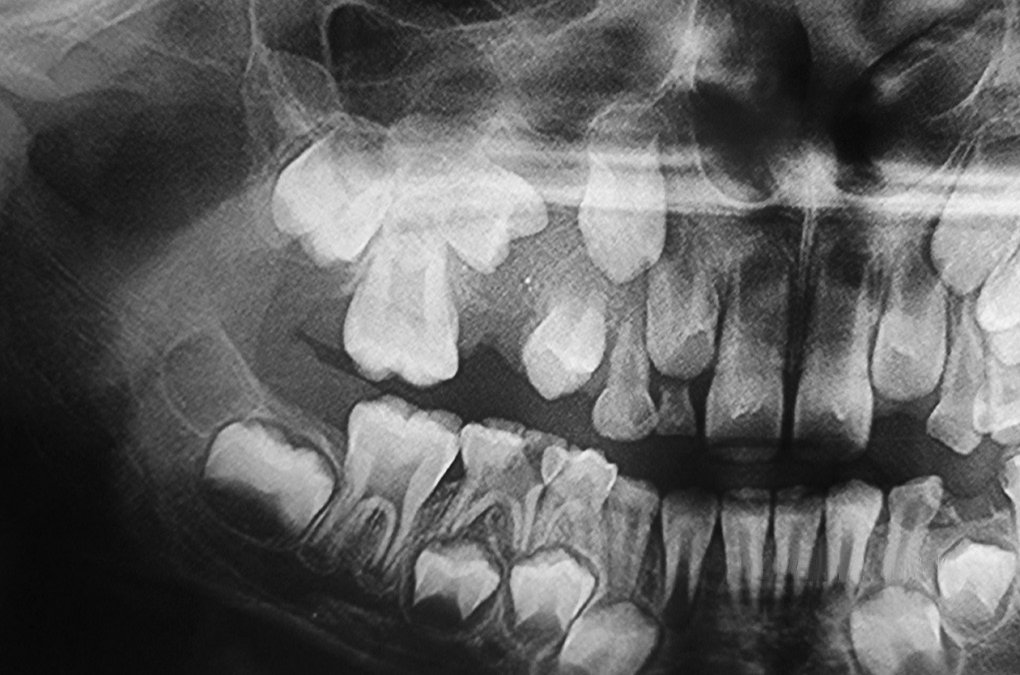
Postoperative OPG (Case 2)

**Fig. 6:**
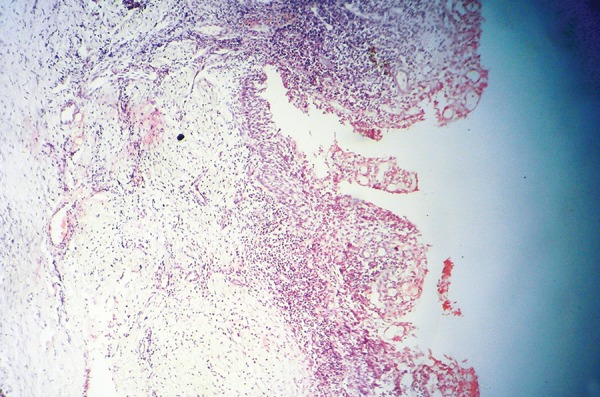
Histopathology (Case 2)

Table 2 shows diagnosis and treatment of these cases. Case 1 involves cystic lesion around developing, severely displaced 34 and the corresponding 74 is noncarious, resorbed (Fig. 1). Surgically enucleated specimen was with lesion attachment at neck of 34. Severely displaced 34 was removed with cystic lining. Figure 2 shows the 4 months postoperative OPG, elimination of cystic cavity and bony trabeculae in its place and erupting 35. Thus, on correlation of clinical, radiological and finding during surgery case of dentigerous cyst of 34. Case 2 was with extensive buccal expansion (Fig. 3). Cystic lesion is around neck of severely displaced 15 as seen in Figure 4, the pre operative OPG. The marsupialization was done and 15 was allowed to erupt. The 3 months postoperative OPG (Fig. 5) shows, elimination of cystic cavity and bony trabeculae of bone formation. The histopathology is seen in Figure 6, cystic lumen covered with proliferation of epithelial lining is indicative of infected cyst. Due to dentigerous relation of cyst with 15 (radiographically) and noncarious corresponding 55, this infected cyst is diagnosed as dentigerous cyst with 15.

**Table 1:** **Table 1:** Case details

*Case no.*		*Age*		*Gender*		*Jaw*		*Deciduous molar tooth*		*Involved premolar displacement*		*Dentigerous position of involved premolar*		*Size of lesion*		*Buccal expansion*	
1		9		F		Mandible		74-resorbed roots, no caries		34-rotated		Yes		15 × 15 mm		Severe	
2		9		M		Maxilla		55-no resorption of roots, no caries		14-Severely displaced		Yes		20 × 20 mm		Moderate	
3		7		F		Mandible		85-caries with pulp involvement, little root resorption		45-in the lesion, not displaced		No		15 × 15 mm		Moderate	
4		6		M		Mandible		85-resorbed roots, no caries		45-severely displaced		Doubtful		15 × 15 mm		Moderate	
5		13		F		Mandible		85-over retained, endodontically treated, roots resorbed		45-little displacement		No		20 × 20 mm		Moderate	

**Table 2:** **Table 2:** Diagnosis and treatment of cases

*Case no.*		*Diagnosis*		*Surgical treatment*		*Follow-up duration*		*Follow-up observation*	
1		Dentigerous cyst of 34		Enucleation, removal of tooth bud 34		2 years		Elimination of cyst, eruption of 35	
2		Dentigerous cyst of 15		Marsupialization		7 months		Elimination of cyst, erupting 15	
3		Radicular cyst of 85		Marsupialization		2 months		Elimination of cyst, erupting 85	
4		Dentigerous cyst of 35 and supernumerary tooth follicle		Enucleation, removal of 75 and supernumerary tooth bud		6 months		Elimination of cyst	
5		Radicular cyst of over retained 85		Enucleation		3 months		Elimination of cyst and erupting 85	

**Fig. 7:**
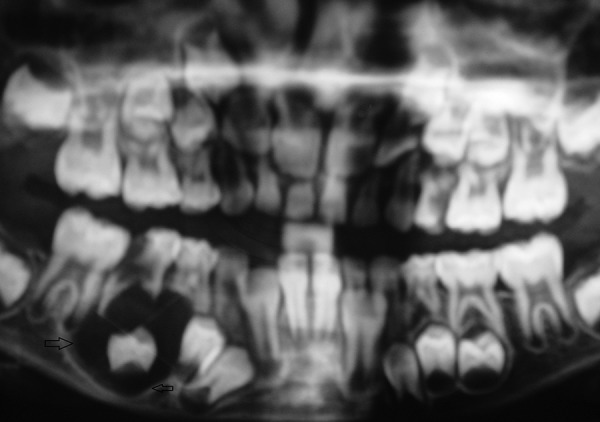
Preoperative OPG (Case 3)

**Fig. 8:**
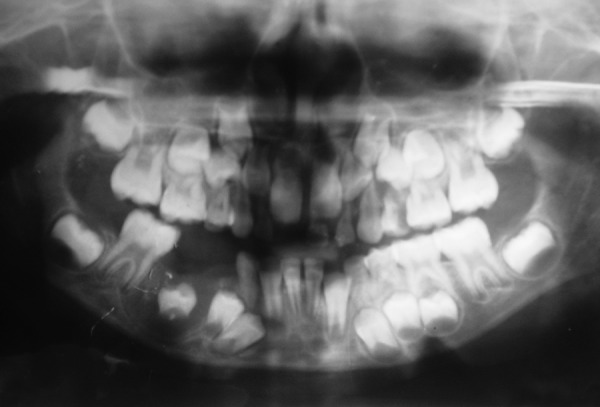
Postoperative OPG (Case 3)

**Fig. 9:**
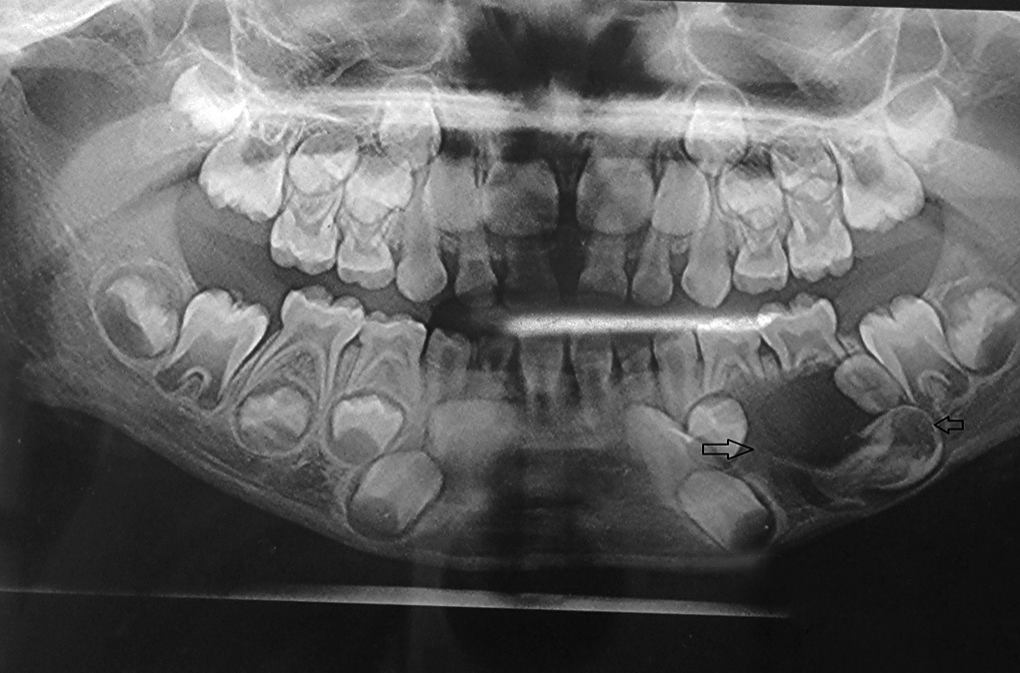
Preoperative OPG (Case 4)

**Fig. 10:**
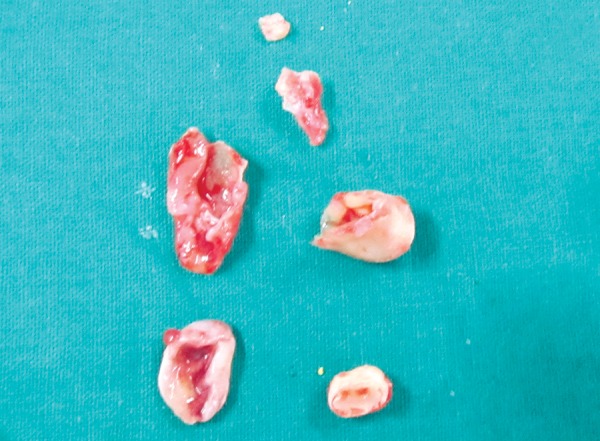
Excised specimen (Case 4)

Case 3 is cystic lesion with carious 85, around its peri-apical and furcation; 45 tooth engulfied in well corticated lesion (Fig. 7). Marsupialization and extraction of carious 85 was performed. Figure 8 shows 1 month postoperative OPG with erupting 45, developing 45 was preserved. This case is diagnosed as radicular cyst as the cyst is typically around the furcation and roots of 85. Case 4 is cystic lesion with severely displaced 34 and 35, and radiopaque ghost image at lower border of mandible, and resorbed intact 74 and 75 (Fig. 9). The case was treated by enucleation of cystic lining, 35 was loose in lining. The supernumerary developi ng tooth cry pt excised with lining was seen as ghost image in OPG. Figure 10 shows excised specimen of cystic lining, 35 and supernumerary. The cyst was involving the supernumerary tooth bud and other teeth 34 and 35 were foating in the lesion as seen on surgical exploration, hence case is diagnosed as dentigerous cyst of supernumerary, displaced 35 was sacrificed.

**Fig. 11:**
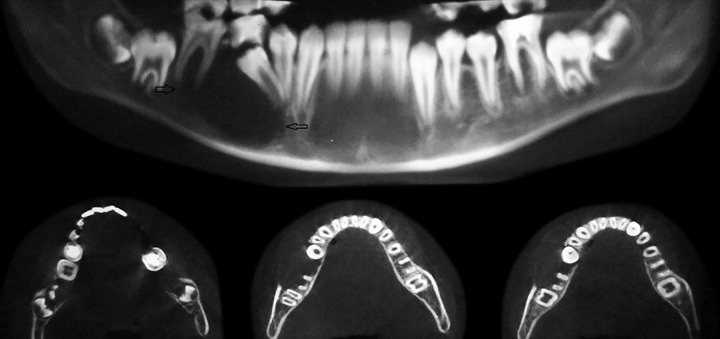
Preoperative CBCT image (Case 5)

**Fig. 12:**
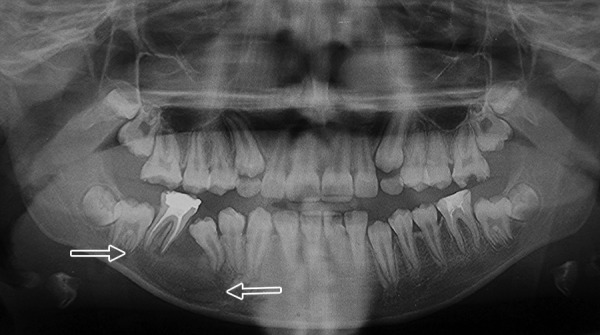
Postoperative OPG (Case 5)

**Fig. 13:**
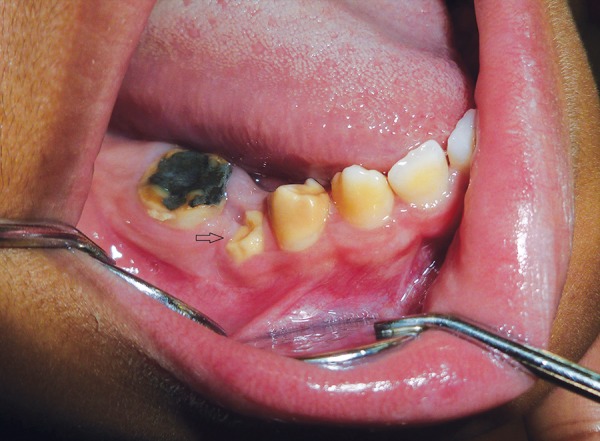
Postoperative clinical image (Case 5)

Case 5 is cystic lesion with endodontically treated, over retained 85. Figure 11 is CBCT image shows developing 45 engulfied in lesion completely. The arrows on panoramic image shows extension of radiolucent lesion from apex of first premolar to the distal root of first molar mesiodistally. There is resorption of roots of 85 and radiolucent lesion extends from the apical region of 85 to the inferior border of mandible, which is intact. The infection may be the reason of absence of cortication in the lesion. The axial image of mandible shows the extension of lesion from first premolar to the roots of first molar. The buccal expansion is evident with discontinuation of buccal cortex. The first molar roots are involved in lesion, it was endodontically treated and extraction of 85 along with marsupialization was performed. The 4 months postoperative OPG shows the elimination cystic lesion, bony trabeculae in its place and 45 erupting along its eruption pathway (Fig. 12). The 45 errupted clinically though in aberrant position (Fig. 13), correction of position is advised. The cyst is typically around periapical region of 85 and the tooth is not in dentigerous relation hence on clinicoradiological correlation case is diagnosed as radicular cyst of 85.

Out of five cases reported here three were labeled as dentigerous cyst and two were labeled as radicular cyst. The histopathological report of all cases is infected dental cyst. Hence, histopathology was not useful for diagnosis. The diagnosis is done on clinical, radiological and surgical correlation. Table 3 shows criteria of case no. 1, 2 and 4 supporting diagnosis of dentigerous cyst of developing premolar tooth bud. Table 4 shows criteria of case no. 3 and 5 supporting diagnosis of radicular cyst of deciduous molar tooth.

## DISCUSSION

Radicular cyst is one which arises from the epithelial residues in the periodontal ligament as a result of infam-mation. The infammation usually follows the death of the dental pulp and cysts arising in this way are found most commonly at the apices of the involved teeth.^1^ Cysts associated with deciduous molars are usually in their periapical region and involve the developing premolar teeth and the patients are bellow 12 to 13 years that is before eruption of premolars or in later age group with over retained molars and delayed eruption of premolars. Shear (1994) has estimated that about 9% of dentigerous and 1 % of radicu lar cysts occur in the first decade of life, while according to Donath (1985) about 4% of dentiger-ous and less than 1% of radicular cysts appear in this life period. In the study of Lustmann and Shear (1985), of radicular cysts with deciduous teeth in 23 cases, the mandible was affected more frequently than maxilla, and the deciduous molars were the teeth most often involved. In nine cases buccal expansion was noticed and in eight cases permanent buds were displaced. The caries was the most common etiological factor.^1^ According to Shear, Radicular cysts of deciduous teeth appear to be rare. Radicular radiolucency related to deciduous teeth tend to be neglected and probably resolve after removal of offending tooth.^1^

**Table 3:** **Table 3:** Criteria of case no. 1, 2 and 4 supporting diagnosis of dentigerous cyst of developing premolar tooth bud

*Dentigerous cyst of developing premolar, 34-case 1: 9 years/female*		*Dentigerous cyst of developing premolar, 55-case 2: 9 years/male*		*Dentigerous cyst of developing premolar 35/supernumerary follicle in mandible-case 4: 6 years/male*	
• Expansion of buccal cortex		• Expansion of buccal cortex		• Expansion of buccal cortex	
• Large size of lesion		• Large size of lesion		• Large size of lesion	
• Dentigerous relation of unicystic radiolucency around crown of displaced premolar on OPG		• Dentigerous relation of unicystic radiolucency around crown of developing premolar on OPG		• Cystic radiolucency is around 75 and the follicle of supernumerary	
• Cyst attached to neck of premolar tooth noted during surgery		• No carious lesion with corresponding deciduous molar		• Both developing premolar tooth buds (74 and 75) are severly displaced	
• No carious lesion with corresponding deciduous molar		• Complete resorption of roots of corresponding deciduous molar		• Root resorption but no carious lesion with deciduous molars	
• No resorption of roots of corresponding deciduous molar		• Severe displacement of involved premolar tooth bud (34) rotated			
• Severe displacement of involved premolar tooth bud (15), above roots of first molar				

**Table 4:** **Table 4:** Criteria of case no. 3 and 5 supporting diagnosis of radicular cyst of deciduous molar tooth

*Radicular cyst of deciduous second molar, 85-case 3:7 years/ male*		*Radicular cyst of deciduous second molar,85-case 5;13 years/ female*	
• Caries involving pulp with second deciduous molar		• Endodonticaly treated over retained second deciduous molar, 85	
• Minimum expansion of buccal cortex		• Minimum expansion of buccal cortex	
• Large size of lesion		• Large size of lesion, involving mesial root of permanent first molar	
• Second premolar tooth bud completely in cystic lesion as seen on OPG		• Second premolar, 45 displaced minimally	
• Premolar tooth bud, 45 not displaced		• Complete tooth (45) in radiolucent lesion and not in dentigerous relationship as seen in OPG	
• At age 7 premolar tooth bud is not having eruptive pressure to erupt in the periapical radiolucency above		• Cystic lining not attached to cervical level of 85	

Periapical cysts in the primary dentition are located around the roots and in the inter-radicular area; this is in contrast to the periapical location seen in permanent teeth. Infammation and prolonged irritation are apparent causes of, and the trigger for, proliferation of the epithelial rests. This explains why cysts are found in conjunction with necrotic or pulp-treated teeth in the primary dentition.^2^ Periradicular cysts that have been described in association with pulpotomized primary molars seem to show specific clinical features: large size, rapid growth, buccal expansion, and displacement of succedaneous teeth.^3^ Dentigerous cyst or follicular cyst is an odontogenic cyst associated with the crown of an impacted, embedded, unerupted or developing tooth. The cyst which encloses the crown of an unerupted tooth is attached to the cervical region of the tooth. It is the second most common type of odontogenic cysts accounting for 14 to 24% of all jaw cysts.^4^ The relationship of cyst development to the mandibular second primary molars cariously affected or pulpotomized has been stressed.^5^ There are two possible explanations: (1) that the mandibular molars have a greater susceptibility to caries and, consequently, are more frequently treated; and (2) that a mandibular second primary molar is more closely associated with its successor's follicle; such association can more easily facilitate the spread of infammation in comparison with other primary teeth.^6^ Shaw et al noted that distinguishing between dentigerous and radicular cysts on histologic grounds is difficult but that a histologic examination may determine whether the lesion is chronic or acute in nature.^6^ In our experience all five cases shows histological features of infected dental cyst (Fig. 5) hence histology was not diagnostic factor. As shown in occlusal radiographs, there are no differences between the two types of cysts with respect to size, buccal bone expansion, or displacement of adjacent permanent teeth.^6^ Periapical radiolucency of primary teeth may be misdiagnosed as a periapical granuloma or a dentigerous cyst of the permanent successor. Many authors prefer the term ‘infamma-tory dentigerous cyst’ for the follicular cyst induced by necrotic or pulp treated primary tooth (da Silva et al, 2002; Benn and Altini, 1996; Aguiló and Gandía, 1998).^7-9^ In the periradicular region of pulpotomized deciduous molars, besides the dentigerous cyst, a glandular odontogenic cyst can develop.^2^

As mentioned by Asian-Gonzélez et al^10^ several differences can been found between the radiological features of periradicular cysts and dentigerous cysts: periradicular cysts are (1) frequently unilocular, although occasionally they are multilocular; (2) they are well-defined radiolucencies, located both periradicularly and inter-radicularly; and (3) the pericoronal space of the underlining permanent tooth appears normal, usually with an intervening total or partially distinct cortical layer of bone. On the contrary, dentigerous cysts: (1)generally have no distinct boundaries between the roots of the primary tooth, and the crown of the underlying succeeding tooth; (2) a single, unilocular, well-defined, radiolucent area encloses each kind of cyst; and (3) a radiolucent area that embraces the permanent tooth bud, ill-defined lesion borders, and arrest of the root development are pathognomonic signs of damaged tooth germs.

Ziccardi et al (1997)^11^ state that, treatment modalities range from enucleation to marsupialization. It is imperative that utilization of a conservative approach to treatment of dentigerous cysts that does not involve, sacrifice of the unerupted permanent tooth whenever possible. However, complete eradication of the cyst should never be compromized for saving the permanent tooth bud. In cases in which the permanent tooth is severely damaged, and hopelessly displaced, complete enucleaton of the cyst to include the permanent tooth bud has been recommended (Brook and Winter, 1975).^12^

Criteria of differentiation as from literature and our study are- radicular cyst- carious or endodonticaly treated deciduous molar; more chances of signs of infection, radiolucency around the roots, involving the furcation as well, complete premolar tooth bud looks engulfied in the lesion on OPG.

### Dentigerous Cyst

No carious lesion with deciduous molar; severe displacement of involved developing premolar as well as adjacent premolar; radiographically and after excision lesion looks typically around premolar crown, even if it is displaced.

### Similar Findings

Expansion of buccal cortex, case presenting as infection, displacement of teeth; aspiration of straw colored fuid unless infected, resorption of roots of deciduous molars, well defined cystic radiolucency with cortication.

## CONCLUSION

The differential diagnosis of periradicular cyst of deciduous molars with carious lesion or endodontically treated can be radicular cyst; or Infammatory denti-gerous cyst of corresponding premolar. The periradi-cular cysts of intact deciduous molars are developmental dentigerous cysts of corresponding premolars. It is a diagnostic dilemma of radicular cyst of deciduous molar *vs* dentigerous cyst of corresponding premolar. This is particularly important for treatment purpose, for preservation of developing premolar and the localized malocclusion developed due to delayed development. We studied the five cases retrospectively for this diagnostic dilemma, and we could set some criteria – three cases of dentigerous cysts and two cases of radicular cyst. Though the sample size of this study is very small, the distinction noted in cases of radicular cyst of deciduous cyst and dentigerous cyst of developing premolar are enough to laid down the criteria for their diagnosis. Although, the studies with larger sample sizes are recommended.
